# Hall’s Solution of Strychnine

**Published:** 1898-09

**Authors:** 


Hall's Solution of Strychnine. ℞.—Strychnia sulph., grs. xvj; sp. vini rect., ℥vij; aq. pura, ℥vij; acetic acid, ʒiv; tr. cardamons co., ℥iss. M. Rub the strychnine to a fine powder in a Wedgewood mortar, then dissolve with the acetic acid; add the alcohol and water and tincture of cardamons, and filter. Each ounce contains one grain of strychnine. This solution is a convenient form for administering strychnine in veterinary practice.

For Uterine Inertia. Pure glycerin introduced into the os uteri, or even into the rectum, during parturition acts promptly in relaxing the rigid os, and also increases the expulsive action of the uterus.

